# Interaction between the Cockayne syndrome B and p53 proteins: implications for aging

**DOI:** 10.18632/aging.100439

**Published:** 2012-02-29

**Authors:** Mattia Frontini, Luca Proietti-De-Santis

**Affiliations:** ^1^ Department of Haematology, University of Cambridge, CB2 0PT, Cambridge, United Kingdom; ^2^ Unit of Molecular Genetics of Aging – Department of Ecology and Biology – University of Tuscia, 01100 Viterbo, Italy

**Keywords:** Cockayne syndrome, p53, senescence, apoptosis, cancer

## Abstract

The CSB protein plays a role in the transcription coupled repair (TCR) branch of the nucleotide excision repair pathway. CSB is very often found mutated in Cockayne syndrome, a segmental progeroid genetic disease characterized by organ degeneration and growth failure. The tumor suppressor p53 plays a pivotal role in triggering senescence and apoptosis and suppressing tumorigenesis. Although p53 is very important to avoid cancer, its excessive activity can be detrimental for the lifespan of the organism. This is why a network of positive and negative feedback loops, which most likely evolved to fine-tune the activity of this tumor suppressor, modulate its induction and activation. Accordingly, an unbalanced p53 activity gives rise to premature aging or cancer.

The physical interaction between CSB and p53 proteins has been known for more than a decade but, despite several hypotheses, nobody has been able to show the functional consequences of this interaction. In this review we resume recent advances towards a more comprehensive understanding of the critical role of this interaction in modulating p53's levels and activity, therefore helping the system find a reasonable equilibrium between the beneficial and the detrimental effects of its activity. This crosstalk re-establishes the physiological balance towards cell proliferation and survival instead of towards cell death, after stressors of a broad nature.

Accordingly, cells bearing mutations in the *csb* gene are unable to re-establish this physiological balance and to properly respond to some stress stimuli and undergo massive apoptosis.

## INTRODUCTION

CSB is a 168 kDa protein that belongs to the SWI/SNF family of chromatin remodelers [1]. It exhibits ATPase activity [2-4] and has conserved helicase motifs [5]. Mutations in CSB gene are often found in Cockayne syndrome (CS), an autosomal recessive, segmental progeroid disorder that affects growth, development and maintenance of a wide range of tissues and organs [6]. Specifically patients exhibit growth failure leading to cachectic dwarfism, severe neurological dysfunction, various somatic changes that resemble aging, gait defects and ocular and skeletal abnormalities. Most patients die during childhood. Its penetrance determines to which severity group (Severe, Moderate, Mild and Adult-onset) the patient belongs; clinical manifestations range from complete disability at birth to mildly affected young adults leading a normal life until the onset of the decline [7]. Despite the fact that the two main genes responsible for CS (csb and csa), were cloned more that 20 years ago [5 and 8] and the function(s) of their products, CSA and CSB proteins, have been subject of intense study, there is currently no cure for this syndrome and treatment is only palliative and directed at alleviating some symptoms. Major biological and clinical features of CS are listed in Table 1.

**Table:**
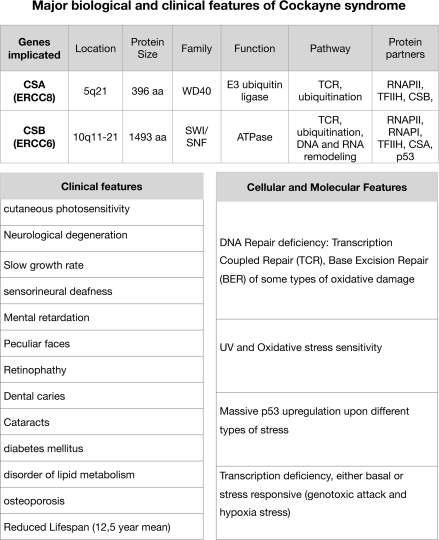


Although we should emphasize that progeroid syndromes are pathological processes and in many aspects they differ from the physiological process of aging (for instance they exhibit only a subset of the symptoms of normal aging), their understanding remain, however, very important to provide new mechanistic insights into normal aging processes. Causative genes can be indeed studied, identifying processes potentially relevant to the mechanism of aging. As an example, the continuous efforts by scientists to understand the cellular and molecular basis of Cockayne syndrome, led them to the discovery of important molecular mechanisms, such as the existence of a peculiar DNA repair pathway devoted to the rapid repair of the transcription-blocking lesions located on the coding sequences of the genome, today known as transcription coupled repair (TCR). As this DNA repair mechanism is impaired in CS patients [9-11] their cells exhibit an extremely delayed recovery of RNA synthesis after ultraviolet radiation (whose lesions on the transcribed strand of genes need to be repaired by TCR), which is responsible of their high UV sensitivity. These findings, besides becoming a diagnostic tool for prenatal diagnosis of CS, offered a strong support to the idea that a major causal factor of aging is the accumulation of DNA damage. Accumulation of DNA damage can also result due to a progressive decrease in the efficiency of the DNA repair systems with age, which may result in progressive cell dysfunction and loss. CSA and CSB proteins have been also associated with other factors and pathways: experimental evidence suggest their involvement in basal and stress responsive transcription as well as in the repair of oxidative DNA damage [12-16]. Defects in these processes are also likely to be responsible for some aging features and the progressive neurological degeneration observed in CS patients. The main aim of this review will be the advances made towards the understanding of the relationship between CSB and p53 proteins and their implications in normal and premature aging.

### p53: suppressing cancer, accelerating aging

The p53 transcription factor integrates different physiological signals in both mammalian and non-mammalian cells. The p53 tumor suppressor is generally considered a protein that is beneficial to the organism. In response to DNA damage, oncogenic activation, hypoxia or other forms of stress, p53 becomes active and triggers multiple specific events, ideally suited to cope with different stress situations. The events triggered by p53 range from a transient (quiescence) to a permanent cell cycle arrest, the latter leading either to cell death via apoptosis or cellular senescence [17]. Recently, p53 has been also implicated in the regulation of autophagy, a lysosomal pathway of cellular self digestion used by eukaryotic cells to deal with diverse physiological functions, including stress adaptation, and protection against neurodegeneration [18]. p53 is a potent tumor suppressors that irreversibly prevents damaged cells from undergoing neoplastic transformation. Accordingly, p53 is one of the most commonly mutated genes in human cancers: being 50% or more of sporadic cancers characterized by somatic p53 mutations. Furthermore, a syndrome linked to germ line mutation of p53, Li-Fraumeni, greatly increases susceptibility to a cluster of early onset cancers [19].

However, both apoptosis and cellular senescence can eventually deplete or inhibit proliferation-competent cells, including progenitor/stem cells, in renewable tissues thus potentially compromising organ homeostasis and accelerating organ degeneration and thus aging [20, 21].

Indeed, the longevity of complex multi-cellular organisms, such as humans, depends on the replenishment of damaged tissues by a small population of adult stem cells able to self renew and be maintained without a significant mutational load. Maintenance of stem/progenitor cell integrity, viability, and self-renewal relies to a great extent on the proper balance between the removal of highly damaged cells via apoptosis and the survival and proliferation of slightly damaged cells, after proper repair. Maintaining this equilibrium, for instance, is particularly important in the tissues where the rate of DNA damage inflicted by free radicals is considerably high.

Recent evidence confirmed that increased p53 activity could, at least under certain circumstances, be disadvantageous for the organism and promote aging. For example, p53-mediated apoptosis and senescence can irreversibly deplete stem/progenitor cell pools from tissues and contribute to organ degeneration. Interestingly, the p53+/m mice with overactive p53 activity displays accelerated aging phenotype, reduced self-renewal and differentiation potential of stem cells and, furthermore it shows an enhanced age associated accumulation of senescent cells compared with wild-type mice [22]. Similarly, the p44+/+ mice carrying extra copies of the hyperactive p53 isoform (p44) shows accelerated aging and reduced number and regenerative potential of neural progenitor cells [23]. Several other knockout and transgenic mice lines that have an increase in p53 activity display premature aging phenotype. In some cases, these aging phenotypes were partially rescued by reduction of the p53 dosage [24]. However, p53 is not necessarily pro-aging *per se*. For instance, additional copies of the p53 gene at its endogenouse locus, in the context of its endogenous genomic location and regulation, result in cancer resistance without aging [25, 26]. Moreover, additional observations underline a positive association between p53 activity and protection against aging or, conversely between decreased p53 activity and aging [27], therefore depicting an unexpected anti-aging activity of p53. Further studies, beside to confirm these observations, showed that p53 does suppress senescence using its transactivation function. Demidenko et al. [28] found that in either p21- or p16- arrested cells, p53 converted senescence into quiescence, a reversible arrest with preservation of proliferation capacity and no senescent morphology. Interestingly the choice between p53-induced senescence and quiescence, in a context of cell cycle blockade, would be mediated by the activity of the mTOR pathway and the strength of the p53 response: while p53 modest activation preserves mTOR activity and therefore results in senescence, strong p53 activation inhibits mTOR and result in quiescence [29-31]. Along these lines it has been suggested that p53 would suppress mTOR through the upregulation of several p53 transcriptional targets, including its negative regulator TSC2 [32]. To this regard, senescence or quiescence have dramatic functional distinctions in cancer and aging; senescence more efficiently halts tumor progression but it is also a stronger inducer of aging.

Together, these results demonstrate that altered (reduced or excessive) p53 activity can be detrimental to the cell and the organism resulting in cancer or premature aging. Therefore it should not be surprising that a complex network of feedback loop mechanisms controls the action of this multi-functional protein.

### The expanding role of CSB/p53 connection

An intriguing connection between CSB and p53 was identified when the two proteins were found to physically interact [33]. The significance of this interaction was totally unclear despite the authors' speculation that a comprehensive binding of p53 to different Nucleotide Excision Repair (NER) proteins, including XPB and XPD, might potentiate the cellular response to DNA damage and result in a more efficient DNA repair. Along these lines, experimental evidences have shown that Li-Fraumeni patients, which bare p53 mutations, display less efficient repair of UV-induced lesions [34, 35]. More recently a model has been proposed whereby CSB would facilitate the sequence-independent chromatin association of p53 [36]. However, it is not clear how this broad and non-specific chromatin enrichment of p53 would help monitoring and maintaining genome integrity, either by scanning for damaged DNA or by helping p53 to find its responsive elements sited on promoters of target genes such as the ones stimulating cell cycle arrest and DNA repair. Ultimately, the function of the CSB-p53 interaction in the context of DNA repair remains elusive.

We have much more information regarding the role of this interaction in the context of transcription. Indeed, we now know that there is a complex connection between these two proteins that coordinate their activities in specific transcriptional programs that regulate cell fate in terms of death or survival after several kinds of stress.

The vast majority of p53 downstream effects are mediated through its intrinsic function as a transcription factor, contributing to the regulation of an expanding spectrum of cellular processes. p53 recognizes its target genes by binding to a consensus response element located proximal to the transcription start site [37]. Besides trans-activating genes whose promoter contain p53 responsive element, we now know that p53 can also trans-repress genes without necessarily binding to their promoters [38]. Trans-repression by p53 may be a result of a competition with others transcription factors for co-activators. Some studies, for instance, demonstrated that p53 and HIF-1 compete for the co-activator p300 [39, 40] and by doing so they antagonize each other until the integration of different signals reaches its balance and the fate of the cell is determined. Similarly, p53 inhibits p300-dependent activation of the TFF2 gene by sequestering p300/CBP away from its promoter [41].

We recently showed that in the absence of CSB, p53 exacerbates this repressive activity. We have shown, indeed, that p300 and CSB compete for p53 binding, with the latter showing a stronger affinity; as a result, p53 transcriptional activity is negatively modulated by CSB [39]. In contrast, the absence of CSB would increase the binding of p53 to p300 causing the stabilization of p53 and the activation of its target genes including the ones involved in the apoptotic commitment. Therefore, the over-activation of p53 response is toxic because this protein titrates away essential transcription factors such as p300. In this instance, CSB has an essential function by interacting with p53 and causing the release of the essential factor p300 from p53. Interestingly, CSB has been shown to recruits p300 at the TCR sites during removal of transcription-blocking lesions [42]. Therefore CSB plays a critical role in cell robustness by down-modulating p53 activity after cellular stress. This role re-equilibrates the physiological response toward cell proliferation and survival after cell cycle arrest and repair, instead that toward cell death. The lack of available p300 in CSB-deficient cells may also explain the general loss of the transcriptional competence that characterizes CSB mutated cells after genotoxic attack. Until recently, the peculiar defect of failing to resume transcription after DNA damage, had been commonly ascribed to the defect in the TCR mechanism responsible for rapidly repairing certain transcription-blocking lesions, located on the transcribed strand of active genes. It is very attractive to speculate that the hyper-activation of the p53 response and the hyper-acetylated state of the p53-regulated promoters would compromise the activations of other promoters contributing to the massive shut down of the transcriptional process. Accordingly, it has been shown that after UV irradiation, neither RNA polymerase II nor the associated basal transcription factors are recruited to the promoters of several genes, housekeeping genes included, around of which histone acetylation is also reduced [43].

Very recently we have also understood how CSB counteract p53 activity by participating to and stimulating its degradation [44]. First, we highlighted that p53 protein levels are permanently up regulated after different types of stress such as oxidative damage and UV irradiation in CS cells. Interestingly, we found that the permanent up regulation of p53 is not obtained at the transcriptional level but it is rather the consequence of a deficiency in its ubiquitination and degradation. This happens because CSB together with CSA, the other protein that when mutated gives rise to CS, is part of an Mdm2 E3 ubiquitin ligase complex that ubiquitinates p53. Noteworthy, p53 binds to the *csb* promoter and transcriptionally controls the expression of the *csb* gene allowing the establishment of a negative feedback loop (where CSB is up regulated) that causes p53 to return to basal levels. It appears that when lacking CSB, this system becomes compromised and unable to sustain and counteract the transient massive up regulation of p53.

**Figure 1:**
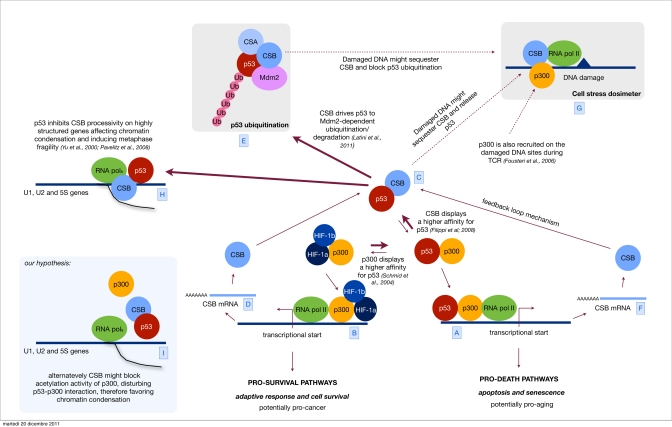
Orchestrating p53 activity Hypoxic stress is known to up regulate both the HIF-1 controlled response and the antagonistic p53 response [**A**]. Sustained p53 activity results in cell demise by promoting the transcription of cell cycle arrest genes, such as p21 and apoptosis-inducing genes, such as Bax [**B**]. Alternatively, HIF-1 can prevent cell death by promoting the transcription of genes that determine adaptive responses and cell survival [**C**]. We have previously showed that CSB, which is also induced by hypoxia [D], interacts with p53 and therefore releases the limiting co-factor p300 from p53. By doing so, CSB negatively modulates the transactivation activity of p53 and up regulates the transactivation of other transcription factor related pathways (in the specific the HIF-1 dependent pathway) therefore re-equilibrating the physiological response toward cell proliferation and survival instead of cell cycle arrest and cell death. [**E**] Recently we have discovered that CSB drives p53 to ubiquitination/degradation, in an Mdm2 dependent fashion, therefore down regulating the cellular levels of p53. CSB is part of a feedback loop where its expression is induced by p53 itself [**F**]. This mechanism helps the cell to find a reasonable equilibrium between the transient up regulation of the p53 response, aimed to temporarily arrest the cell cycle and potentiate the DNA repair mechanism, and its down modulation in order not to reach the point of no-return that would trigger apoptosis. Others have established that after genotoxic stress (UV, oxidative stress) CSB is also recruited to DNA damaged sites [**G**], we propose a model in which CSB, besides acting in DNA repair, may act as a sort of dosimeter in order to modulate either the transcriptional activity of p53 (p53-p300 interaction) or its degradation. Indeed, high levels of DNA damage would sequester CSB at the damaged sites thus blocking its function in the down modulation of p53 activity and degradation. Parallel recruitment of p300 by CSB favors the accessibility of the damaged site to the repairosome and potentially inhibits the transcription (at least the p300 related) contributing to the transient shut down of the transcription in response to DNA damage. [**H**] Finally, the interaction CSB/p53 has been suggested also to play a role in metaphase fragility. p53 inhibits CSB-processivity affecting chromatin condensation of highly structured genes, such as U1, U2 and 5S, resulting in metaphase fragility [**I**]. We propose an alternative hypothesis, where CSB might favor chromatin condensation on these sites by disturbing p300/p53 interaction and chromatin acetylation.

Accordingly, CSB appears to function as a factor that controls the levels of p53 and may regulate the fate of its activity. We propose a model in which a low level of DNA damage or any stress that the cell may somehow be able to deal with give rise only to a minor p53 response aimed to transiently arrest the cell cycle in order to allow the cell to repair DNA damage and/or deal with other kind of stress. In this case CSB, whose expression is directly controlled by p53 itself, after a while would drive the cells to re-establish the basal level of p53. Instead, in circumstances of high stress, including heavy DNA damage, p53 levels would remain high, ultimately driving the cell to die. What may be the mechanism that eventually pauses the function of CSB in limiting p53 activity? It has been demonstrated that CSB is also involved in DNA repair and in fact CSB co-localizes with the repairosome on the damaged sites [42, 36]. We speculate that depending on the amount of the DNA damage, CSB might be sequestered away from p53. According to this hypothesis the sequestration of CSB to the damaged sites would function as a dosimeter of sorts that would fine-tune the activity of p53 and more importantly decide the fate of the cell (Figure 1).

Additionally, it has been previously described that p53 is able to inhibit the function of CSB leading to metaphase fragility. Based on the observation that loss of CSB or overxpression of p53 induces metaphase fragility of four loci each containing tandem repeat of genes for abundant small RNAs, such as U1, U2 and 5S RNA [45], Weiner's group propose that CSB could function as an elongation factor for the transcription of these highly structured RNAs. In the absence of functional CSB, RNA polymerase II would stall on the U1, U2 and 5S genes, locally blocking metaphase chromatin condensation and thereby causing metaphase fragility. More recently the same group suggested a second scenario in which the lack CSB would inhibit the disassembly of the transcription complex on these highly transcribed genes, during metaphase, therefore affecting chromatin condensation [46]. Having confirmed an interaction between CSB and p53, they also propose that activated p53 would sequester, modulate or inactivate scarce CSB protein thus phenocopying the effect of mutations inactivating it. According to this hypothesis, p53 would act as antagonist of CSB. Though this hypothesis is very attractive, authors did not show the presence of CSB at these specific loci. More importantly it is not clear what would be the biological role of p53-induced inhibition of CSB. In the light of our recent data we cannot exclude that CSB instead, either by interacting with and/or by ubiquitinating p53, might protect these sites from the perturbing effects of p53 recruitment [47]. The absence of CSB would both enhance the expression of p53, as we described above, and would make these sites more accessible to p53. Alternatively, CSB might disturb p53/p300 interaction on these sites affecting chromatin acetylation therefore favoring their condensation. Further studies will be required to discriminate between these hypotheses.

### The role of CSB toward p53 in counteracting aging

The “antagonistic pleiotropy” theory of aging suggests that aging results from genes with positive effects on fitness early in life but with negative effects later on. Thought recent revisitations of p53 functions are challenging the antagonistic pleiotropy model [48], the p53 gene has been shown to influence lifespan and fitness, in a similar way. In response to stresses of different nature, the p53 protein is stabilized and triggers a number of processes including senescence and apoptosis in order to prevent damaged cells to undergo neoplastic transformation. Senescence and apoptosis slowly but inexorably deplete the tissues of cells therefore promoting aging. However, being p53 action wisely regulated by a series of feedback loop mechanisms, its impact on aging is postponed and the time of onset correlates roughly with the lifespan of the organism. The deregulation of p53 and the consequent enhanced apoptotic response, in the absence of the CSB protein, gives rise to pronounced cell fragility when cells are exposed to stress of broad nature and can potentially explain the multiple degenerative problems including central nervous system degeneration, premature arterio-sclerosis, progressive joint deformities and loss of subcutaneous fat, in CS patients. A very interesting work performed *in vivo* by the Cleaver's group [49] associated the degeneration of Purkinje cells with the up regulation of p53 in double knock-out CSB^−/−^/XPC^−/−^ mice. Unfortunately, this work, although very illustrative, in terms of p53/neurodegeneration relationship, highlights that the neural phenotype exhibited by the single knockout CSB^−/−^ mouse is much less severe than the one displayed by the human CS patients and raises serious questions about using the mouse as model to study the molecular basis of CS, at least for what concerns neurodegeneration.

On the other hand, there is mounting evidence that apoptosis and cellular senescence, associated to the elevated expression of p53, have a part in premature aging disorders such as Parkinson's, Alzheimer's and Huntington's diseases [50].

### CSB and p53, a relationship in promoting cancer

The reason for the absence of increased cancer incidence in CS patients, despite of the defect in DNA repair, is currently unknown. This is a peculiarity of CS patients since another human syndrome, *Xeroderma pigmentosum* (XP), characterized by a defect in the same DNA repair pathway, displays a 1,000-fold increase in cancer incidence [51]. Moreover strong evidence have been provided for the anti-neoplastic potential of the CSB defect in a background of p16^Ink4a^/p19^ARF^ or p53 tumor suppressor deficiency [52]. Therefore, lack of CSB seems to result in cancer protection. The defect in DNA repair without any increase of the mutation load can be explained only by assuming that the lack of CSB forces damaged cells into the apoptotic pathway and therefore cancer could not take advantage of the DNA repair defect. Or in other words: the mutator phenotype potentially conferred by the lack of the DNA repair function of CSB is recessive while the cancer resistance, also conferred by the lack of CSB, is dominant. The increased levels of apoptosis that takes place after many kinds of stress in the absence of CSB, led us to speculate that this could be the main reason. In the context of the CSB^−/−^ associated transcriptome, p53-transactivated transcriptional pathways that lead cells to die would prevail over the pro-survival pathways. Failure of the hypoxia adaptive response in the CSB^−/−^ environment is very illustrative and it is likely to be not the only one [39]. In this regard, it is well known that the expression of oncogenes, such as Ras or Myc, elicits p53 response [53]; perhaps lack of CSB would inexorably lower the apoptotic threshold of the cells. Very interestingly, we should highlight that this increased apoptotic process, linked to CSB deficiency, could be also p53-independent. Two publications, in fact, highlighted an enhanced apoptotic potential correlated to the deficiency of CSB even in p53 knock-out mice [52] or cells possessing a mutated p53 protein unable to perform transactivation activity [54]. However, having these data originated in different rodents further studies are necessary to shed more light to the role of CSB in triggering apoptosis in a p53-independent way.

Another aspect of CSB functionality as a coactivator might help to explain why CSB cells display cancer resistance. The boosting of certain transcriptional programs, on which cancer cells relay, may be unachievable in cells lacking CSB, for instance. It is well know that beside DNA repair, CSB is strongly involved in other DNA metabolic activities including RNA pol I, II and III transcription [4, 12, 13, 14, 16 and 55]. It is also likely that a reduced activity of the above mentioned activities might limit the “upgrading” of the cancer cells. Interestingly our recent analysis of a panel of several tumor tissues and cell lines highlighted overexpression of CSB and CSB silencing leads to cell death (unpublished).

Therefore, the concept of a balance between cellular aging and cancer susceptibility maintained by levels of p53 and the role played by CSB protein in this context is very attractive.
